# Aerosol emission rates from breathing, speaking, and speaking with a surgical mask

**DOI:** 10.3389/fpubh.2026.1833687

**Published:** 2026-07-13

**Authors:** Carl Firle, Oliver Stier, Anke Steinmetz

**Affiliations:** 1 Division 4 Hazardous Substances and Biological Agents, Unit 4.II.2 Bioaerosols, Federal Institute for Occupational Safety and Health, Berlin, Germany; 2 Voline Systems GmbH, Berlin, Germany; 3 Institute of Medical Psychology, University Medicine Greifswald, Greifswald, Germany

**Keywords:** aerosol emission, aerosol measurement, surgical masks, speaking, chamber study, leakage, particle emission rate, respiratory aerosols

## Abstract

Transmission risk assessment of airborne diseases such as COVID-19 requires a detailed understanding of respiratory aerosol generation and emission during activities such as breathing and speaking, as well as the influence of protective devices such as surgical face masks. We measured integral aerosol emission rates from *n* = 44 participants during breathing, speaking, and speaking while wearing a surgical mask, with each condition performed for 20 min. All measurements were conducted in a closed 20 m^3^ cabin under standardized initial conditions in an operating theater environment. Particle emission rates were log-normal distributed across participants. Median emission rates were 60 particles per second (P/s) for breathing (*n* = 35, IQR 24–152 P/s), 135 P/s for speaking (*n* = 41, *IQR* 56–327 P/s), and 278 P/s for speaking with a surgical mask (*n* = 29, *IQR* 157–492 P/s). Under the investigated conditions, wearing a surgical mask did not reduce total aerosol emission and was associated with a higher emission rate (median ratio 1.34, 95% CI [1.04–1.75], two clearly separated observations omitted). This observation may be explained by a combination of effects. First, the present measurements reflect total particle emission into the environment and therefore capture system-level behavior, including leakage, rather than material filtration efficiency. Second, previous studies have reported that mask use can alter respiratory mechanics, which may contribute to changes in aerosol emission. These findings may be relevant for assessing long-range transmission risk under comparable environmental conditions. At the same time, they do not contradict the established effectiveness of surgical masks in reducing droplet-mediated short-range transmission.

## Introduction

Airborne infections like SARS-CoV-2 can reach high infection rates by aerosol transmission. Outbreaks in choirs suggest that long-range transmission is the main route of infection and only explicable by aerosol transmission rather than droplet transmission (1, 2). A current evaluation of online risk assessment tools proves good quality for outcome parameters while emphasizing the need for more qualified particle emission rates and mask efficiency values (3–6). Cluster-randomized trials showed mixed effects depending on face mask type on infection rate (7, 8).

Aerosol generation during breathing and speaking has been investigated extensively in recent years, particularly in the context of airborne disease transmission (9–16). Experimental studies have demonstrated substantial inter-individual variability in particle emission rates, as well as a strong dependence on vocal activity and speaking intensity. In particular, particle emission has been shown to increase with vocalization and loudness, while particle size distributions are typically dominated by submicron particles originating from the respiratory tract (9, 10, 13).

In parallel, numerous studies have examined the filtration performance of face masks. Under controlled laboratory conditions, mask materials—including those used in surgical masks—have been shown to exhibit significant filtration efficiencies for particles in the submicron size range (11, 12). However, such measurements typically characterize the intrinsic filtration properties of the mask material and do not account for leakage flows that occur under practical wearing conditions.

More recent investigations have emphasized the importance of considering mask performance as a system-level phenomenon that includes not only material filtration but also fit, leakage, and altered airflow patterns. In particular, it has been shown that masks can substantially modify the flow field of exhaled air, redistributing particle transport rather than simply reducing emission (14, 16). These effects complicate the direct translation of material filtration efficiency into real-world emission reduction.

Existing experimental approaches to aerosol emission can be broadly divided into local measurements near the mouth or mask surface and integral measurements of total particle emission into a defined volume. Local measurements are well suited to characterize filtration properties and near-field effects but may underestimate total particle release in the presence of leakage. In contrast, chamber-based methods capture total emission independent of flow direction and thus provide a direct measure of the effective particle source strength (17–21).

Recent studies illustrate the value of near-field and transport-oriented approaches. In a portable isolation chamber study, CO_2_ tracer measurements and CFD simulations were used to evaluate how local negative-pressure extraction near a patient can reduce contaminant dispersion in a room (22). In a related CFD and experimental study, different mask materials were compared with respect to porosity, air permeability, pore size, and droplet transport, showing that mask structure and leakage strongly influence near-field aerosol dispersion (23). Such approaches are well suited to investigate local flow fields, material-related mask performance, leakage pathways, and the interaction between exhaled particles and ventilation or extraction systems.

The chamber-based method used in the present study addresses a complementary question. It does not resolve the local direction of exhaled flow or individual leakage pathways. Instead, it quantifies the total particle emission accumulating in a closed volume and therefore captures the net outward emission of the human–mask system independent of the direction in which particles leave the mouth or mask. This distinction is particularly relevant for surgical masks, where leakage and flow redirection may cause local measurements at a single position to underestimate total particle release.

The measurement of aerosol emission rates based on particle accumulation in a closed volume allows assessment of the net effect of mask use on total particle emission, taking into account both filtration and leakage effects. By focusing on paired comparisons within individuals, our study isolates the effect of mask use from inter-individual variability and provides a complementary perspective to existing studies that primarily assess material filtration efficiency or near-field measurements.

The particle emission rate depends on physical activity. During breathing at rest participants seem to emit particles at the lowest emission rates of around 100 particles/second (P/s) (24, 25). Other studies found lower particle emission rates of approximately 10 P/s (26, 27). Speaking results in higher breathing activity and thus higher particle emission. Most of the studies report around 500 P/s for speaking (24, 25, 28, 29), but also lower particle emission rates of 30–60 P/s have been reported (27, 30–32). Childrens' particle emission rates were assessed likewise and ranged up to 100–150 P/s for speaking and loud speaking (33). Further increase in breathing activity results in even higher particle emission rates, as during wind instrument playing, 1,500 P/s (20, 34, 35), and singing, 1,000–6,000 P/s (2, 28, 36). High physical exercise results in similar particle emission rates of 1,000–1,300 P/s (26, 27, 37).

Measurement of particle emission rates from speaking with a mask is more difficult and less results have been published. Asadi et al. (38) detected median particle emission rates for breathing as low as 0.31 P/s. Wearing a surgical mask reduced the particle emission to 0.06 P/s and wearing a KN95 mask resulted in 0.07 P/s. The particle emission rate from speaking was 2.77 P/s and from speaking with a surgical mask 0.18 P/s. Wearing a KN95 mask during speaking was found to result in 0.36 P/s. Contrary to hypothesis, wearing an unwashed single layer t-shirt mask resulted in increased emission of 0.61 P/s during breathing and 16.37 P/s during speaking. Based on those data, outward efficiency values were calculated: 90% for surgical mask, 74% for KN95 respirator. As limitation the authors highlighted that they did not detect the total of emitted particles vanishing in non-outward direction, e.g., top or sides. The values reported therefore likely underestimate the particle emission rates.

The latter problem of leakage has also been described by Szczepanska et al. (31). They detected lower aerosol concentrations in front of the surgical mask during breathing and speaking. The filtration efficiency was calculated to 80–87% with increasing efficiency for larger particle sizes > 2 μm.

A recent study by Toghraei and Romanic (21) measured particle concentrations resulting from different breathing activities of six participants sitting in a closed cabin. The experimental setup and data evaluation procedure were similar to that of Firle et al. (20). The authors found the highest aerosol concentrations from male participants speaking without mask. Using a surgical mask during speaking reduced human aerosol emission for both sexes to a level comparable to breathing without mask. The lowest aerosol emission was obtained from breathing with a mask. The reduction effect of a mask on particle emission was estimated as 63 %. Regarding the particle size distribution, the study found a superposition of two lognormal distributions with modes between 0.200–0.235 and 1.97–2.45 μm, respectively.

A different methodological approach was chosen by Bennett et al. (39). They injected sodium chloride particles of 0.05 μm diameter behind the mask worn by participants. At a distance of 60 cm a condensation particle counter recorded particle concentrations under different conditions (breathing in rest, reading out loud, coughing) persistent during 3 min. The authors report containment efficiencies of 43.2 % for breathing at rest with a procedure face mask and 41.0 % for speaking with the same mask.

In conclusion, the measurement of particle emission rates at different levels of breathing activity during mask wearing is experimentally challenging. To circumvent the difficulties previously encountered we designed a novel experimental setup to measure the total respiratory particle emission from breathing, speaking and speaking with a surgical mask. The aim of our study is reassessment of the efficiency of surgical masks for the reduction of outward aerosol emission.

The novelty of this study lies in quantifying total aerosol emission under masked vs. unmasked conditions using a chamber-based approach that captures both filtration and leakage effects.

## Methods

### Study design and participants

The core structure of our study and the design of experiment have been published by us in Firle et al. (20). In this cross-sectional study we assessed particle emission rates from wind instrument playing. As control tasks the musicians had been asked to breathe and speak for 20 min, the latter one, with and without wearing a surgical mask. The present publication focusses on observations during the control tasks, regarding the effect of using surgical masks. Standard commercially available surgical masks were used (type II R/EN 14683, model Lanhine Surgical Mask blue L, LOT FM-BL, REF FM-BL-1, 2020-04-10, Zhejian Lanhine Medical Products Ltd., China), consisting of a three-layer non-woven structure (spunbond–meltblown–spunbond).

Further volunteers had been recruited for the control tasks, resulting in a total of *n* = 44 individuals. Not all participants performed the task speaking with surgical mask (*n* = 29). Speaking tasks were standardized by instructing participants to read aloud continuously from a predefined text (the beginning of Hermann Hesse's “Der Steppenwolf”, in German) for 20 min at a natural, clearly audible speaking level. While no quantitative sound pressure level was imposed, participants were asked to maintain a consistent vocal effort comparable to typical reading aloud in everyday situations. The relatively long duration of the single tasks was chosen in order to obtain a sufficient signal-to-noise ratio of the calculated emission rates.

To minimize variability unrelated to respiratory particle generation, participants were instructed and supervised during mask placement. Surgical masks were worn according to standard medical practice, ensuring typical real-world fit conditions. The experimental design does not aim to eliminate all leakage pathways but instead captures the effective emission under realistic usage conditions, including potential leakage at mask edges. This approach reflects actual mask performance in everyday scenarios rather than idealized filtration efficiency.

Inclusion criterion for the non-musician group was major age and exclusion criteria included acute or severe chronic respiratory diseases and symptoms of a SARS-CoV-2 infection. Ethical approval was given for the preliminary study by the ethics committee of the Greifswald University Hospital (reference number BB 131/20). Every participant gave informed written consent. The study was registered retrospectively on the German Clinical Trials Register (http://www.drks.de) as No. DRKS00023336. The research was performed in accordance with relevant guidelines, regulations, and the Declaration of Helsinki.

### Setup and data analysis

Since the study was designed to assess unaided vocal emission under controlled conditions no external amplification (e.g., microphones) was used. Exhalation flow rates were not measured or controlled, as the study focuses on integrated particle emission rather than underlying flow dynamics. Our experimental design focuses on measuring the total aerosol outward release under controlled conditions rather than resolving the underlying physiological mechanisms. Hence, individual anatomical factors influencing aerosol generation were not assessed in this study.

The measurement setup, experimental procedure, and data evaluation are described completely in our previous publication (20). To ensure reproducibility and clarity, the measurement principle is described here explicitly. The experimental approach is based on quantifying the total number of aerosol particles emitted by a participant in a closed environment.

Aerosol emissions are quantified in terms of particle number concentrations rather than particle mass because the particle number is conserved during transport from the respiratory tract to the measurement location, whereas particle mass and size may change due to evaporation and hygroscopic effects. Considering particle number rather than mass facilitates comparison of emission rates across studies and avoids uncertainties associated with assumptions about particle composition and density.

The analysis focuses on particles with diameters below approximately 6.6 μm, which remain airborne for extended periods and are therefore relevant for long-range transmission. Larger particles are subject to rapid gravitational settling and were rarely detected under the present experimental conditions.

The participants performed all tasks one by one in a hermetically closed cabin with 20 m^3^ volume, in which all particles emitted by the participant accumulate over time. Under these conditions, the temporal increase in aerosol particle concentration reflects the net emission of particles into the enclosed air volume.

Given sufficiently homogeneous mixing of air and particles within the chamber, the particle emission rate can be derived from the rate of increase in particle concentration. Specifically, the emission rate is equal to the slope of the concentration–time curve multiplied by the chamber volume. This approach allows quantification of total aerosol emission independent of directional airflow or leakage effects, which are known to limit the accuracy of local sampling methods.

**Figure 1:**
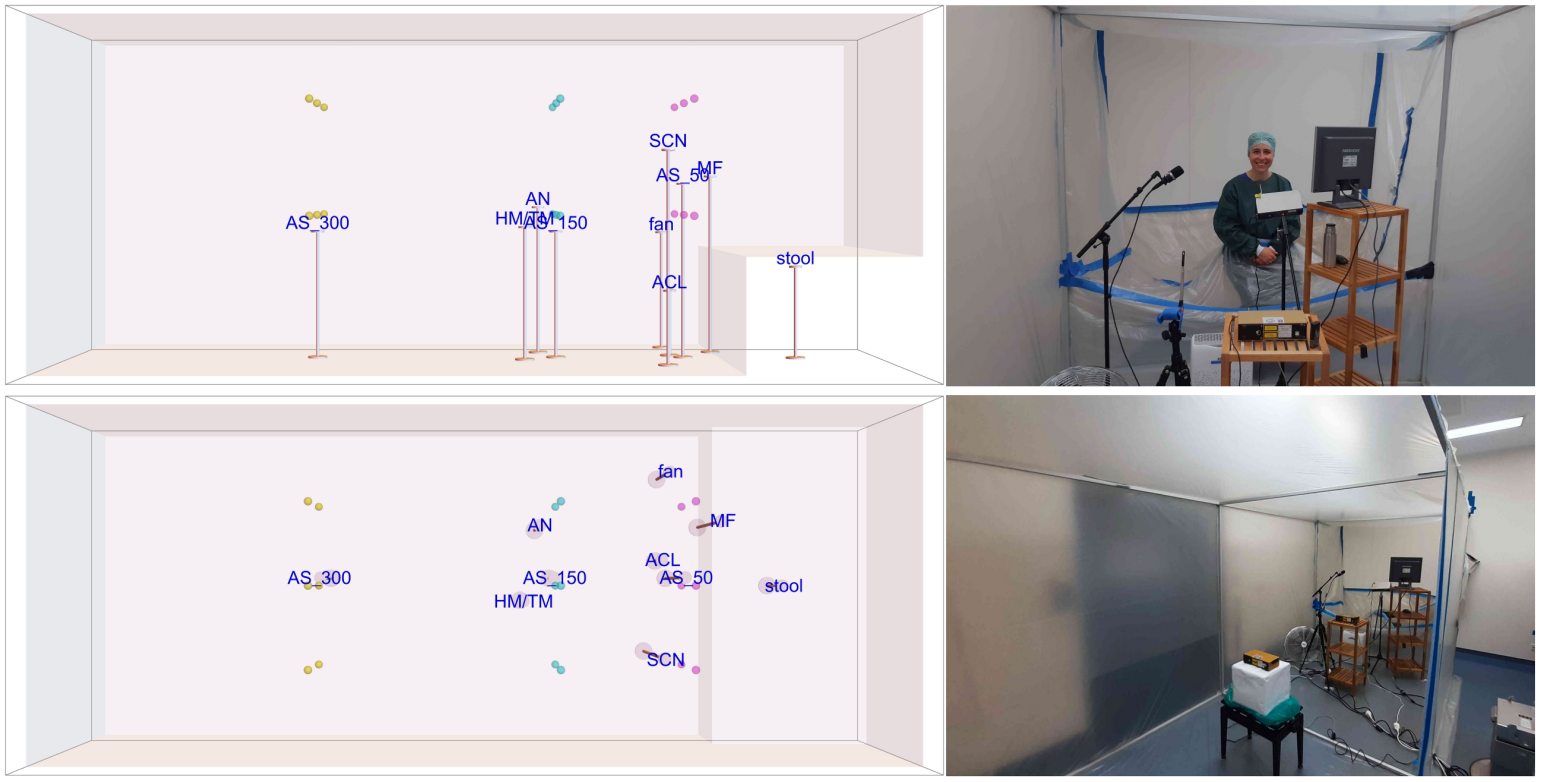
Schematic representation of the experimental setup showing the sealed chamber in front and top view, participant position (stool), and locations of the aerosol spectrometers (AS), anemometer (AN), hygrometer/thermometer (HM/TM), microphone (MF), computer screen (SCN), and air cleaner (ACL), scales in cm. The participant gave her informed consent to publication of the photograph.

The measurement cabin was installed in an operating theater, see Figure 1. All participants were clothed in surgical gowns and hoods to suppress particle release from their clothes and hair. In the breaks between subsequent tasks the cabin was always ventilated with purging air to recondition the particle concentration to the background level in the operating theater (approximately 20 particles per liter). Environmental conditions within the operating theatre were controlled and remained stable throughout the measurements following a normal distribution. Room temperature was in average 23.4 ± 0.4 °C, relative humidity 39 ± 6 %, and air pressure 1,005 ± 5 hPa.

The sealed chamber design ensures minimal influence of external ventilation or airflow patterns during measurements. While this setup differs from real-world environments, it is specifically intended to isolate the particle emission process itself from environmental dilution or transport effects. Consequently, the measured emission rates represent source strengths under controlled conditions and can be used as input parameters for transmission risk models, where environmental factors such as ventilation and air mixing are considered separately.

We measured the temporal increase of the spatially uniform aerosol concentration in the cabin surrounding the participants during the 20 min period of task activity. The aerosol particle number concentrations were measured using three optical particle counters simultaneously (Grimm aerosol spectrometers 1.109 and 11-D), positioned at distances of 0.5 m, 1.5 m, and 3 m from the participant. This spatial distribution of sensors allows verification of homogeneous particle distribution within the chamber.

Homogeneous particle mixing within the chamber was assessed by comparing the time-dependent concentration signals recorded simultaneously at the three spectrometer positions. As described in detail in our previous methodological work, Firle et al. (20), concentration curves obtained at the different sampling locations were largely consistent after temporal averaging, indicating sufficient mixing of airborne particles within the measurement volume. The present analysis therefore uses concentration values averaged over all three instruments. This procedure reduces the influence of local concentration fluctuations and avoids dependence on a single sampling position. Air velocity inside the chamber was very low during measurements, with mean values below 0.02 m/s, indicating the absence of relevant directed airflow. Thus, the measured concentration increase can be interpreted as an accumulation of particles in a nearly stagnant, enclosed volume rather than as a result of local transport toward individual sampling positions.

**Figure 2:**
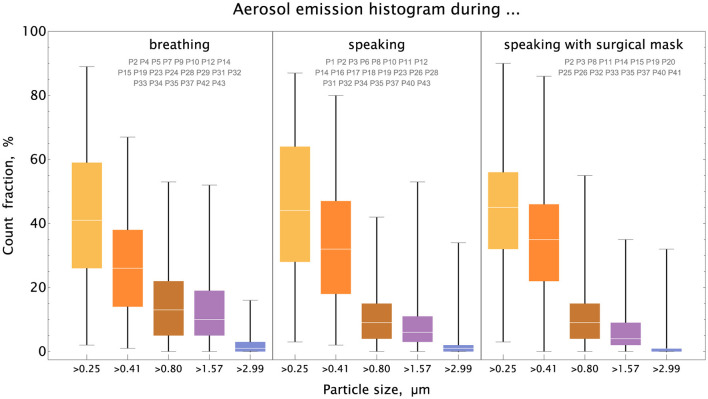
Particle size distributions as box whisker charts for the three different tasks. No significant differences between the tasks are observed.

According to manufacturer specifications, the Grimm instruments provide single-particle counting with validated sizing and counting performance consistent with ISO 21501-1 calibration procedures. Counting efficiency remains 100% below particle concentrations of 100,000 particles per liter (P/l). During the present measurements concentrations remained below 1,000 P/l, thus well below the coincidence loss threshold. The instruments operate on the principle of laser light scattering and detect individual particles within 31 log-equidistant particle size bins covering the total particle size range 0.25–35 μm. For improvement of the signal to noise ratio the default bins were merged into the five bins shown in Figure 2 (>0.25, >0.41, >0.80, >1.57, >2.99 μm) during data post processing. Larger particles were rarely detected. Measurements were recorded at 6-s intervals, corresponding to a sampling volume of 120 ml air per measurement cycle (sample flow rate 1.2 l/min ± 5%). Due to the discrete nature of particle counting during finite volume sampling, counting precision and reproducibility at low particle concentrations are limited by counting statistics and follow a Poisson distribution. A profound discussion is provided in the Supplement of Firle et al. (20). All instruments were factory-calibrated according to manufacturer specifications and operated within their specified operating conditions.

To further improve measurement accuracy, particle concentration data were averaged over time and across all three instruments. The resulting average concentration values were used to estimate the sustained particle emission rate of the participant during the respective task via linear regression. The dominant source of measurement uncertainty arises from the intrinsic random fluctuation of particle counts and was quantified using a bootstrap-based approach to estimate confidence intervals of emission rates. These individual confidence intervals are stated along with the expectation values. Emission rates were not treated as single point estimates but as distributions characterized by confidence intervals, thereby capturing both measurement noise and variability in the regression procedure. Accordingly, they are displayed in the Figures below by their means and standard deviations. The reported confidence intervals reflect both measurement uncertainty and inter-individual variability in emission rates.

The smallest air concentration of particles that is safely distinguishable from zero by this procedure is 5 P/l for particle diameters < 2.5 μm. The smallest emission rate detectable by the described procedure was 25 particles per second (P/s).

Particle loss to chamber surfaces was considered as a potential source of underestimation of absolute emission rates. In the sealed chamber, ventilation-related particle loss during measurements was negligible because there was no intentional air exchange with the surrounding room. The remaining loss mechanism is deposition of airborne particles on chamber walls and other surfaces. In our previous methodological analysis, Firle et al. (20), this process was modeled as a first-order surface deposition loss term. Based on measurements under comparable chamber conditions, the corresponding loss coefficient was estimated to be small, and the resulting correction for the surface absorption effect in our setting mainly causes a moderate increase in absolute emission rates *Q*:


$$\begin{array}{l} Q\to \;1.02\;Q+33\text{P}/\text{s} \end{array}$$ (1)

For the purpose of the present study, this correction was not applied because it does not affect the comparative interpretation of the data. The correction acts as a positive monotonic transformation of the measured emission rates and therefore preserves their ordering across tasks and participants. Consequently, if one measured emission rate is higher than another before correction, it remains higher after correction. In addition, the magnitude of the wall-loss correction is small relative to the estimation uncertainty of most individual emission rates. Wall deposition may therefore lead to a moderate underestimation of absolute emission rates, but it is not expected to generate or reverse the observed differences between experimental conditions.

Evaporation of respiratory aerosol particles is known to affect measured particle sizes (40) while leaving particle count numbers unaltered due to solid hygroscopic residue. Therefore, evaporation has no effect on our results or conclusions which are entirely based on count rates.

Data evaluation and statistics were all performed with *Wolfram Mathematica*, Version 14.1, Champaign, IL (2024). All data sets are available in this Supplement.

## Results

Group characteristics are reported in Table 1. We did not target any specific distribution for age or gender. We obtained a uniform age distribution and a 59 % female portion. None of the participants had acute respiratory symptoms, as required for inclusion. Eighty-four percent of the participants were non-smokers.

**Table 1:** Group characteristics.

Characteristic	Median (*IQR*, min–max range) or *n* (%)
Age [years]	45 (28–56, 20–68)
Gender identity female: male: divers	26: 17: 1 (59: 39: 2)
Weight [kg]	70 (58–80, 50–95)
Height [cm]	172 (165–180, 156–203)
BMI [kg/m^2^]	23 (21–25, 18–38)
Respiratory disease	3 reported allergic asthma (no acute exacerbation)
Nicotine consumption none : currently : previously	37 : 4 : 3 (84 : 9 : 7)

**Figure 3:**
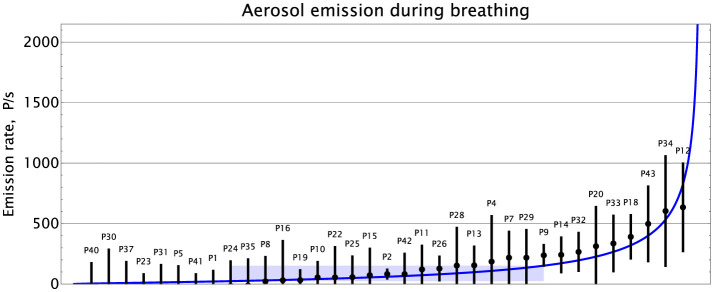
Distribution of aerosol emission rates (particles per second, P/s) during breathing for 35 participants, those excluded having overall negative as-measured emission rates. Black dots indicate maximum likelihood estimates and black vertical bars indicate credibility ranges. The blue curve is the cumulative distribution function (CDF) of the log-normal (4.10, 1.37) distribution obtained by fitting the data (*p* < 0.001). The blue box is the interquartile range, *IQR* 24–152 P/s, median is 60 P/s. The “*P*” numbers above the data points label the participants.

Figure 3 displays the as-measured particle emission rates during calm breathing. Black dots indicate the maximum likelihood point estimates of the emission rates and black vertical bars indicate the credibility ranges. There are several measurements where the point estimation gave unplausible, negative values while the credibility range extends to the physically plausible, positive range. These are the leftmost data points whose dots lie outside the clipped plot range. Measurements where not even the credibility range contains positive emission rates have been discarded as technically unsuccessful. The lengths of the error bars—the magnitudes of the credibility ranges—vary significantly, meaning that the individual measurements produced results of different accuracy. Any statistical analysis neglecting these individual accuracies of the measurements would be prone to misleading conclusions. Therefore, we apply only methods who allow to include the accuracy information associated with each data point.

To determine the likely statistical distribution of the emission rates we have sorted them in ascending order by their point estimation values. Note that this ordering is not affected by particle absorption on surfaces and would not be altered by applying the correction Equation 1. The *k*-th value in an ordered sample of *n* random variates is most likely the *p*-quantile of the underlying distribution, with $p\;=\;\frac{k}{\left(n+1\right)}$. The ordered emission rates thus present an approximation of the cumulative distribution function (CDF) of their distribution. We explored various physically plausible distributions using the Kolmogorov-Smirnov test and found the lognormal distribution to be the parsimonious one. The blue curve in the plot shows the CDF of the log-normal (4.10, 1.37) distribution which was obtained as least-squares fit to the data points taking into account their error bars (41). The interquartile range (IQR) of that distribution is displayed by the blue rectangle, *IQR* is 24–152 P/s and median is 60 P/s.

**Figure 4:**
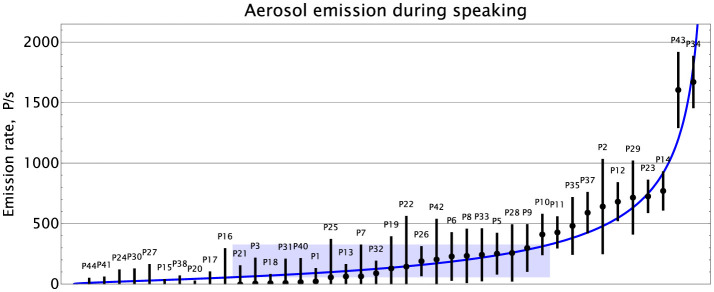
Distribution of aerosol emission rates during speaking without mask for 41 participants, those excluded having overall negative as-measured emission rates. Black dots indicate maximum likelihood estimates and black vertical bars indicate credibility ranges. The blue curve is the CDF of the log-normal (4.91, 1.31) distribution obtained by fitting the data (*p* < 0.001). The blue box is the *IQR* 56–327 P/s, median is 135 P/s. The “*P*” numbers above the data points label the participants.

**Figure 5:**
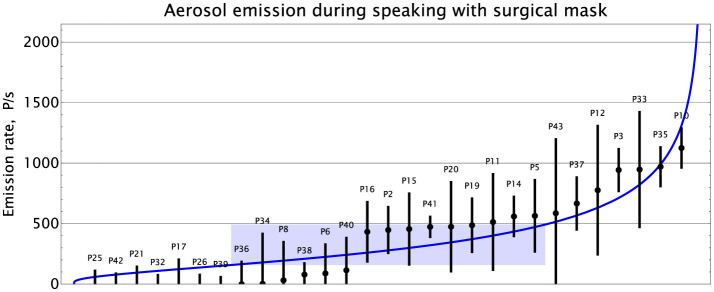
Distribution of aerosol emission rates during speaking with surgical mask for 29 participants, those excluded having overall negative as-measured emission rates. Black dots indicate maximum likelihood estimates and black vertical bars indicate credibility ranges. The blue curve is the CDF of the log-normal (5.63, 0.85) distribution obtained by fitting the data (*p* = 0.32). The blue box is the *IQR* 157–492 P/s, median is 278 P/s. The “*P*” numbers above the data points label the participants.

The particle emission rates from speaking are evaluated and displayed in the same manner in Figure 4: Median 135 P/s, *IQR* 56–327 P/s, distribution lognormal. Other than expected, the particle emission rates were even higher when speaking with a surgical mask: Median 278 P/s, *IQR* 157–492 P/s, as displayed in Figure 5. The distribution is still potentially lognormal, but the χ^2^ value is larger (*p* = 0.323) than for the other two distribution fits (*p* < 0.001).

The medians in Figures 4, 5 suggest that emission rates increase when participants put on a surgical mask. We do not apply a rank sum test to determine whether the median difference is statistically significant because such a test can only take into account the point estimates of the emission rates, whereas their estimation uncertainties would have to be neglected. Since the univariate single-task samples in Figures 4, 5 also differ in size, 41 vs. 29, they shall not be used for estimation of the emission rate ratio between speaking with and without a mask.

**Figure 6:**
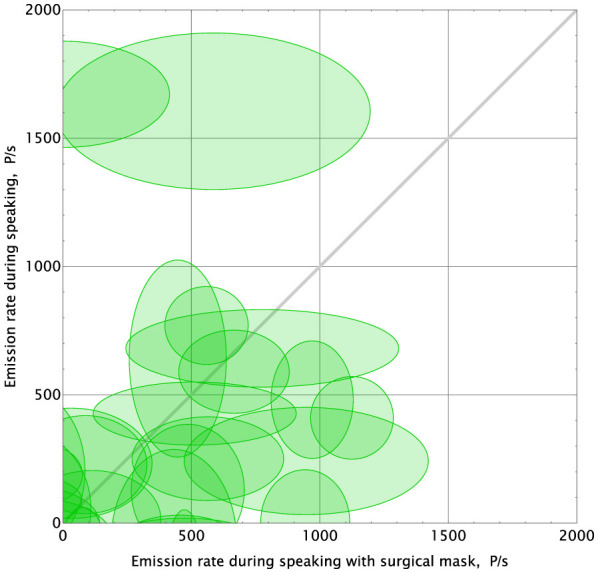
Aerosol emission rates for speaking with and without mask. The grey line indicates equal emission rates during both tasks. There are two clearly separated observations having substantially higher particle emission rates from speaking without mask than from speaking with mask (P34 and P43), as opposed to the majority of participants who produced somewhat greater emission rates when speaking with a surgical mask.

To evaluate the difference between speaking with and without mask we plot the credibility ranges of the particle emission rates from speaking with and without mask for the 27 applicable participants in Figure 6. There is a significant cluster of participants producing higher particle emission rates from speaking with surgical mask than from speaking without mask, represented by the green ellipses below the grey diagonal line. Note that the wall loss correction Equation 1 would magnify the ellipses by 2% and shift them parallel to the grey diagonal by 47 P/s, which would not change the above observation.

The green ellipses in Figure 6 indicate, for each participant, the joint probability density function (PDF) of the bivariate normal distribution of the posterior likelihood of a given pair of emission rates from speaking with and without mask, respectively. The likelihood is greater at, or near, the center of an ellipse than on its boundary. Therefore, certain ratios between the emission rates with and without mask are more likely than others, given the present point estimates (ellipse centroids) and separate credibility ranges (ellipse semiaxes) calculated from the measurements. The fact that our measurement results are represented by PDF reflects the estimation uncertainty of emission rates as calculated from aerosol concentration time series. The most likely ratio, overall, maximizes the product of likelihoods for all participants included. The median and corresponding confidence intervals of this ratio were used to summarize the overall effect across participants.

Figure 6 shows a strictly paired analysis between masked and unmasked conditions, thus avoiding problems related to confounding parameters or unequal sample sizes as in Figures 4, 5. Only individuals who performed both tasks belong to the bivariate sample, so we compare here the same individuals across the masked and unmasked conditions. Statistical robustness and representativeness are unaffected by the existence of further individuals who performed one of the tasks only. This paired analysis controls for inter-individual variability in baseline emission strength and allows isolation of the effect of mask use.

Two participants (P34 and P43) show emission rate ratios that are markedly separated from the main cluster of observations in the bivariate distribution (Figure 6). This separation is evident as a large geometric distance from the cluster of the remaining participants and exists independent of assumptions regarding the underlying causes of the deviation. Since no experimental or physiological explanation for these deviations could be established, the distinction between the main cluster and the two separated observations is based on the geometry of the bivariate paired data.

This analytical treatment of P34 and P43 follows standard concepts from robust statistics and anomaly detection. In these frameworks, clearly separated observations are not automatically treated as invalid data. Instead, the main body of the data is characterized while separated observations are retained and interpreted separately, particularly when the data suggest latent heterogeneity or more than one response pattern (42–44).

**Figure 7:**
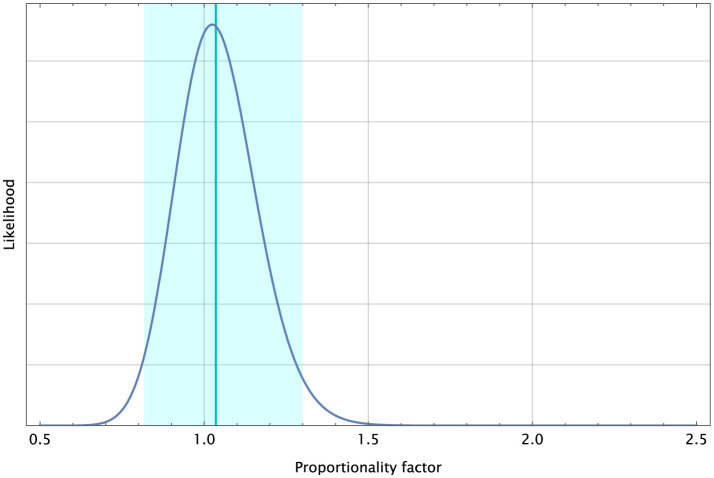
Likelihood function of the paired emission-rate ratio, defined as aerosol emission rate during speaking with a surgical mask divided by aerosol emission rate during speaking without a mask. All paired observations, including P34 and P43, were included in this analysis. The blue rectangle indicates the 95% *CI* [0.82–1.30], and the blue vertical line indicates the median ratio of 1.04. Thus, when all paired observations are included, the estimated overall ratio is close to unity. This result reflects the opposing response pattern of the two clearly separated observations compared with the main participant cluster shown in Figure 6.

**Figure 8:**
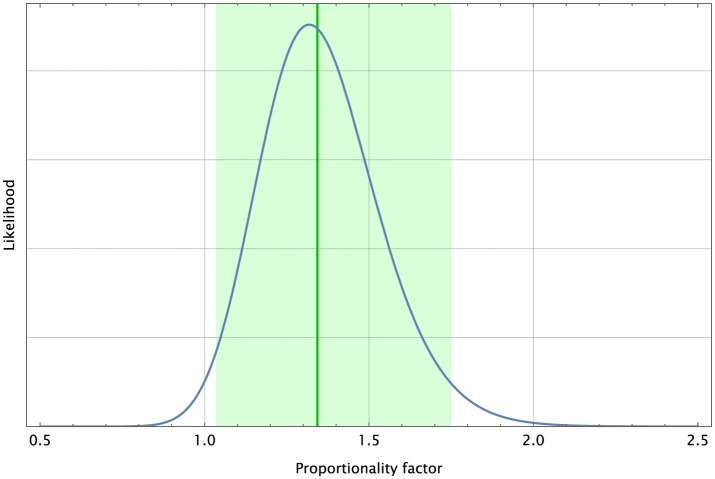
Likelihood function of the paired emission-rate ratio, defined as aerosol emission rate during speaking with a surgical mask divided by aerosol emission rate during speaking without a mask. This main-cluster analysis excludes the two clearly separated observations P34 and P43. The green rectangle indicates the 95% *CI* [1.04–1.75], and the green vertical line indicates the median ratio of 1.34. In the main participant cluster, speaking with a surgical mask was associated with higher total aerosol emission than speaking without a mask (expectation value is the factor 1.36).

Hence, we estimate the ratio between the emission rates obtained with and without mask for two different sets of participants: First, including all participants from Figure 6 (result shown in Figure 7) and second, excluding the two separated observations P34 and P43 (result shown in Figure 8). In either case we calculate, first, the total likelihood function of the emission rate ratio for the respective set of participants and subsequently the median and confidence interval (CI).

According to the first estimation, including all observations, the median ratio is 1.04 with the 95% *CI* [0.82–1.30]. The median emission rate ratio is close to unity (Figure 7), indicating no overall effect of wearing a mask. However, the mask appears to have no effect because the opposite effects observed for the main cluster of participants and the two separated observations cancel.

When the latter are excluded, the data of the remaining 25 participants indicate increased emission during masked speaking, with a median ratio of 1.34 and a 95% *CI* [1.04–1.75], see Figure 8. On average, 36 % greater emission rates are estimated, indicating that surgical mask use was associated with amplified aerosol emission during speaking in the main participant cluster.

Conversely, applying the same likelihood-based ratio estimation to the observations P34 and P43 alone yields an expected emission-rate ratio of 0.2 with a 95% *CI* [0.01–0.57]. This result is consistent with the initially expected direction of a mask effect, namely reduced outward emission during masked speaking, and therefore supports the interpretation that P34 and P43 represent realistic observations rather than invalid or technically suspect measurements. P34 and P43 are not separated from the remaining observations merely by large univariate values, but by an opposite response to mask use. At the same time, they are internally coherent. We therefore treat these two observations as a minority response pattern.

The remaining 25 participants form the main data body for which the likelihood-based ratio estimate characterizes the dominant majority response pattern. Since the individual uncertainty ellipses differ in size, this estimation also accounts for heteroskedastic measurement uncertainty across participants.

Taken together, the paired data are better described as comprising two response patterns rather than a single homogeneous response across all participants. The comparison with Figure 7 demonstrates that the full-sample estimate is strongly influenced by the coexistence of these opposing response patterns. Reporting both the full-sample estimate and the main-cluster estimate therefore avoids both the unjustified exclusion of separated observations and the masking of the dominant response pattern by a small, oppositely responding subgroup.

While we found an increase in total particle emission, the particle size distribution remains unaltered during all three tasks, see Figure 2.

## Discussion

The present study identifies a trend-level effect indicating that, on average, aerosol particle concentrations measured in the environment of a speaker are higher when a surgical mask is worn compared to speaking without a mask. In the analyzed paired subset, this corresponds to an average increase in emission rates by approximately 36 %. Importantly, this effect is not uniform across all individuals, as some participants exhibited reduced emission rates when wearing a mask. Overall, our data do not support a consistent particle retention effect of surgical masks under the investigated conditions.

Aside from this observation, the remaining findings of the present study—such as the magnitude and variability of aerosol emission rates during breathing and speaking, as well as the predominance of submicron particles—are in line with previously published results (24, 25, 27–29). These concordant observations serve to validate the experimental approach and indicate that the study is consistent with established knowledge, except for the specific effect related to mask use, which was not anticipated and, to our knowledge, has not been reported in this form before. A possible physiological interpretation of this observation is discussed below, but it remains indirect because respiratory mechanics and leakage flow dynamics were not measured in the present study.

In contrast, the implication of increased aerosol emission for long-range transmission risk follows directly from established principles of airborne infection dynamics. The probability of infection in indoor environments is determined by the inhaled dose of aerosol particles, which depends on the emission rate of the source, the duration of exposure, and the dilution conditions in the environment (3). Higher emission rates lead to increased inhalation doses for exposed individuals. This relationship is well described by dose–response models of airborne infection and has been confirmed in multiple epidemiological analyses of indoor transmission events (1, 2). Therefore, an increase in emitted aerosol particle number is expected to translate into a corresponding increase in long-range transmission risk under otherwise comparable conditions.

Our particle emission rates from breathing and speaking are consistent with previously published values for breathing (100 P/s) and speaking (30–500 P/s). Thus, we assume valid data acquisition and a reliable methodological approach, measuring increasing aerosol concentrations in a sealed clean-air cabin. This experimental setup allows to circumvent problems that had been discussed earlier, i.e., particle leakage escape from measurement area (38). Moreover, we were able to detect sufficient particle counts resulting in emission rates above detection limit. Asadi et al. (38) report particle emission rates valuing nearby zero. We assume that such values are below detection threshold and thus too low, as also discussed by the authors themselves. Although residual wall deposition and imperfect mixing cannot be excluded entirely in any chamber-based measurement, these effects are expected to affect absolute emission-rate estimates rather than the paired comparison between masked and unmasked speaking conditions.

Regarding the particle size distribution, most of the particles are below 1 μm for all tasks and confirm previous study results (9, 10, 24, 45–50). The constant particle size distribution supports the hypothesis that particle formation is independent from the task itself but depends on the task-specific physiological breathing pattern. Previous studies have shown that breathing patterns influence particle generation in the respiratory tract (50).

The emission rates of all tasks likely have a lognormal distribution, as also found in a study of the emissions during voice assessment and therapy tasks (51) and a study assessing aerosol emissions using a surgical mask (21). In agreement with previous studies our data indicate that breathing emits least respiratory particles, median 60 P/s (*IQR* 24–152 P/s). Particle emission from speaking is higher, median 135 P/s (*IQR* 56–327 P/s).

An unexpected result is the finding that the average particle emission rate is higher from speaking with surgical mask than from speaking without mask across a sample of 25 individuals: The emission rate ratio between the masked and unmasked conditions is most likely 1.3, median 1.34, 95% *CI* [1.04–1.75], hence, the measured increase of aerosol emissions during mask use is unlikely accidental. While this contradicts several previous studies it does not conflict with established filtration physics and is consistent with physiological studies.

The term “filtration efficiency” requires clarification in the context of the present study. In the technical literature, filtration efficiency typically refers to the fraction of particles removed by the filter material itself under controlled conditions, often measured in a directional and material-specific manner. These measurements usually characterize the intrinsic properties of the mask fabric and do not account for leakage flows around the mask.

In contrast, the present study does not measure material filtration efficiency but rather the total aerosol emission into the surrounding environment during exhalation. This quantity reflects the combined effect of filtration through the mask material and leakage of exhaled air around the mask edges. In practical use, especially for surgical masks, such leakage flows are known to be substantial and can dominate the overall particle release.

The observed increase in emitted particle number during masked speaking therefore does not contradict reported filtration efficiencies of mask materials. Instead, it indicates that under realistic wearing conditions, the effective outward particle retention is governed primarily by leakage rather than by the intrinsic filtration properties of the mask material.

For this reason, the present results should be interpreted as a measure of effective outward emission under real-use conditions rather than as a direct assessment of mask filtration efficiency in the technical sense. This interpretation is supported by direct visualization studies of aerosol dispersion during mask use. Kniesburges et al. (52) demonstrated, using aerosol cloud imaging during professional singing, that surgical masks substantially alter the direction and spatial distribution of the exhaled aerosol flow rather than simply eliminating aerosol release. In their measurements, aerosol escaped through leakage pathways at the cheeks and nose bridge and was redirected toward the near-field region around and above the head. At the same time, forward dispersion was reduced. These findings illustrate that the outward performance of a surgical mask is not determined by material filtration efficiency alone, but by the combined effects of filtration, fit, leakage pathways, and flow redirection. A chamber-based approach, as used in the present study, is therefore well suited to quantify total outward particle emission independent of the direction in which particles leave the mask.

The present study did not experimentally distinguish between respiratory particles and potential particles originating from the surgical mask material itself, such as fiber shedding induced by mask deformation during speaking. Zhao et al. (53) summarize in Table 1 studies investigating particle release from disposable surgical masks. They predominantly report fibrous or microfiber-like particles with sizes >100 μm originating from non–woven polypropylene materials similar to those used in the present study. Since the particle size distributions observed here remained largely consistent across tasks (Figure 2), no clear indication of a dominant contribution from larger mask-derived fibers was observed. A separate experimental isolation of mask-derived particle release under physiologically realistic speaking conditions was beyond the scope of the present work. Therefore, a contribution of mask-derived particles to the observed increase in aerosol emission cannot be fully excluded.

The observed increase in particle emission during masked speaking may be tentatively explained by mask-induced changes in respiratory mechanics and breathing pattern. Surgical masks have been shown to increase airway resistance in healthy volunteers during steady-state exercise (54). In a systematic review and meta-analysis, mask use was associated with altered respiratory rate and ventilation, with stronger effects reported for FFP2/N95 respirators than for surgical masks (55). These findings indicate that mask wearing can modify respiratory mechanics, even if standard clinical parameters or perceived exertion are only mildly affected.

Studies investigating respiratory function during mask use, including those reporting minimal changes in standard clinical parameters, often focus on cardiovascular outcomes (56) or oxygen saturation and/or subjective breathing comfort (57) rather than aerosol generation.

A physiological link between respiratory mechanics and aerosol generation is further supported by recent data showing that indices of airway resistance and reactance obtained by impulse oscillometry correlate with aerosol particle emission (37). In addition, it has been shown that human aerosol emission per breath depends non-linearly on breathing volume (58) and inspiratory duration, with higher breathing volumes and shorter inspiratory durations leading to substantially increased particle emission (50). This finding provides direct experimental support for the concept that changes in breathing pattern can alter aerosol generation. Increased respiratory effort and larger tidal volumes can be associated with enhanced aerosol particle generation in the respiratory tract (49).

Taken together, these studies provide a plausible physiological framework for the observed increase in total particle emission during masked speaking. However, this interpretation remains indirect because breathing volume, respiratory rate, airway resistance, expiratory airflow velocity at the mouth, and leakage jet velocities at the mask edges were not measured in the present study. Air velocity measurements in the present study refer to residual air motion within the chamber and were used to verify the absence of relevant directed airflow in the measurement volume. They do not represent measurements of expiratory airflow velocity or leakage pathways. Thus, respiratory flow dynamics and leakage pathways were not resolved experimentally in the present study.

The facial seal quality of the masks in our study was representative of randomly selected participants wearing a surgical mask under physician supervision. All particles escaping from the human–mask system contributed to the measured chamber concentration. This experimental condition is relevant for assessing real-use outward emission from surgical masks and may even overestimate particle retention compared with unsupervised everyday mask use. Under these conditions, surgical masks did not produce a consistent reduction in total outward aerosol emission.

Our findings therefore suggest that surgical masks with low effective outward particle retention and substantial leakage may fail to reduce, and under some conditions may even increase, total aerosol emission into the surrounding environment. This does not contradict their established role in reducing droplet-mediated short-range exposure. However, it indicates that surgical masks should not be assumed to provide reliable protection against long-range aerosol-mediated transmission. The measured emission rates should be interpreted as source-term data relevant for airborne-transmission models, rather than as direct estimates of infection risk.

## Conclusion

Our data confirm that speaking emits more respiratory particles than breathing.

Under the investigated conditions, wearing a surgical mask was associated with higher total aerosol emission during speaking. The measured emission rates are otherwise consistent with previously reported values.

This observation may be tentatively explained by a combination of effects. The present measurements capture total particle emission into the environment, including leakage and potentially minor contributions from mask-derived particles, rather than material filtration efficiency. In addition, previous studies have reported that mask use can alter respiratory mechanics, which may contribute to changes in aerosol emission.

Emitted particles may reach the surrounding environment and are therefore relevant for assessing long-range transmission under comparable conditions.

The particle size distribution remained consistent across tasks, in line with previous findings indicating that aerosol generation is influenced by physiological breathing patterns.

These results should not be interpreted as a general statement against mask use in public health. Rather, they indicate that surgical masks may have limited effectiveness for reducing long-range aerosol source strength under the investigated conditions, while their established role in reducing droplet-mediated short-range exposure remains unaffected. For public-health interpretation, the present emission rates should therefore be considered source-term data for airborne transmission models rather than direct estimates of infection risk. Further studies are required to systematically investigate the effects of different mask types, fit conditions, and respiratory activities on total aerosol emission under controlled and real-world conditions.

## Data Availability

The original contributions presented in the study are included in the article/Supplementary material, further inquiries can be directed to the corresponding author.
